# The relative importance of large problems far away versus small problems closer to home: insights into limiting the spread of antimicrobial resistance in England

**DOI:** 10.1186/s12916-017-0844-2

**Published:** 2017-04-27

**Authors:** Tjibbe Donker, Katherine L. Henderson, Katie L. Hopkins, Andrew R. Dodgson, Stephanie Thomas, Derrick W. Crook, Tim E. A. Peto, Alan P. Johnson, Neil Woodford, A. Sarah Walker, Julie V. Robotham

**Affiliations:** 1 The National Institute for Health Research (NIHR) Health Protection Research Unit in Healthcare Associated Infections and Antimicrobial Resistance, University of Oxford, Oxford, UK; 2 Nuffield Department of Medicine, University of Oxford, Oxford, UK; 3 National Infection Service, Public Health England, Colindale, London, UK; 4 Public Health Laboratory, Public Health England, Manchester Royal Infirmary, Manchester, UK; 5 Department of Microbiology, Central Manchester University Hospitals NHS Foundation Trust, Manchester, UK; 6 Microbiology Department, University Hospital South Manchester, Manchester, UK; 7 NIHR Biomedical Research Centre, Oxford, UK

**Keywords:** Antimicrobial resistance, Infection prevention and control, Regional coordination, Screening strategies, Carbapenemase-producing Enterobacteriaceae, Hospital network

## Abstract

**Background:**

To combat the spread of antimicrobial resistance (AMR), hospitals are advised to screen high-risk patients for carriage of antibiotic-resistant bacteria on admission. This often includes patients previously admitted to hospitals with a high AMR prevalence. However, the ability of such a strategy to identify introductions (and hence prevent onward transmission) is unclear, as it depends on AMR prevalence in each hospital, the number of patients moving between hospitals, and the number of hospitals considered ‘high risk’.

**Methods:**

We tracked patient movements using data from the National Health Service of England Hospital Episode Statistics and estimated differences in regional AMR prevalences using, as an exemplar, data collected through the national reference laboratory service of Public Health England on carbapenemase-producing Enterobacteriaceae (CPE) from 2008 to 2014. Combining these datasets, we calculated expected CPE introductions into hospitals from across the hospital network to assess the effectiveness of admission screening based on defining high-prevalence hospitals as high risk.

**Results:**

Based on numbers of exchanged patients, the English hospital network can be divided into 14 referral regions. England saw a sharp increase in numbers of CPE isolates referred to the national reference laboratory over 7 years, from 26 isolates in 2008 to 1649 in 2014. Large regional differences in numbers of confirmed CPE isolates overlapped with regional structuring of patient movements between hospitals. However, despite these large differences in prevalence between regions, we estimated that hospitals received only a small proportion (1.8%) of CPE-colonised patients from hospitals outside their own region, which decreased over time.

**Conclusions:**

In contrast to the focus on import screening based on assigning a few hospitals as ‘high risk’, patient transfers between hospitals with small AMR problems in the same region often pose a larger absolute threat than patient transfers from hospitals in other regions with large problems, even if the prevalence in other regions is orders of magnitude higher. Because the difference in numbers of exchanged patients, between and within regions, was mostly larger than the difference in CPE prevalence, it would be more effective for hospitals to focus on their own populations or region to inform control efforts rather than focussing on problems elsewhere.

**Electronic supplementary material:**

The online version of this article (doi:10.1186/s12916-017-0844-2) contains supplementary material, which is available to authorized users.

## Background

Responsibility for the control and prevention of antimicrobial resistance (AMR) traditionally lies with individual healthcare institutions as they are perceived to be the main source of transmission. The rationale is that hospitals that do not invest in infection prevention and control (IPC) will have a higher prevalence of resistant bacteria. However, hospitals may receive patients who acquired resistant bacteria during a stay in another hospital, as a patient’s colonisation with resistant bacteria is often associated with previous hospital stay [1, 2]. Through these shared patients, hospitals are regularly exposed to resistance from surrounding hospitals, as well as those further afield or overseas.

The movement of resistant bacteria between hospitals through shared patients has several important ramifications. First, the exposure to resistance from surrounding hospitals means that AMR rates of neighbouring hospitals are interdependent, particularly for hospitals that exchange many patients – high rates in one hospital will result in multiple introductions to the other [3]. Second, hospitals receiving many transferred patients, such as major tertiary referral centres, are at an increased risk of AMR introductions [3]. A large proportion of these shared patients are indirectly transferred, with a stay in the community between discharge from one hospital and admission to the next [4]. Third, investments in IPC in one hospital affect its neighbouring hospitals through a reduction in the threat of AMR introductions and exposure for their ‘neighbours’ [5]. This also implies that preferential investments in hospitals that share many patients with others are more effective than investments in all hospitals uniformly [6].

The inter-hospital spread of AMR is not just a theoretical concept, as evidenced by numerous hospital network studies. In particular, the number of patients a hospital receives from other hospitals, i.e. the weighted in-degree, correlates well with incidences of healthcare-associated infections such as *Clostridium difficile* [7] and methicillin-resistant *Staphylococcus aureus* (MRSA) [8]. Furthermore, advanced molecular methods that allow bacterial populations to be compared between hospitals show, for instance, that hospitals exchanging a large number of patients have similar MRSA populations, with comparable frequencies of sequence types [9] and low levels of genome sequence diversity [10].

The realisation that introductions from other hospitals may affect within-hospital rates of AMR has prompted the formation of IPC policies that take account of hospital transfers. The Dutch Working group on Infection Prevention [11] recommends that patients who have previously been admitted to a hospital with a ‘current MRSA outbreak’ should be considered as high risk and be placed in pre-emptive isolation on admission. More recently, Public Health England (PHE) published similar advice concerning carbapenemase-producing Enterobacteriaceae (CPE) in their CPE toolkit for hospitals [12], recommending that patients admitted within the last 12 months to a hospital with a ‘known CPE problem’ should be isolated and screened. The focus on previously admitted patients to specific hospitals as a high-risk category is intended to avoid the need for universal admission screening since, on a relative scale, a greater number of colonised patients will be identified per 1000 patients screened.

The ability of control strategies categorising hospitals as high risk to avoid unidentified introductions (and hence potential onward transmission) in other hospitals is unclear, as the number of patients that require screening (and the potential introductions associated with them) depends on the number of hospitals on the high-risk list, their AMR prevalence and the number of patients shared with each recipient hospital. The goal of this study was to investigate the relationship between the structure of hospital networks (due to patient movements) and potential AMR introductions in order to inform how hospitals should address the increasing number of AMR threats likely to arise over the next decade. We used the current, most pressing AMR concern of CPE as an exemplar for the possible regional differences in prevalence. We tracked patient movements, estimated local and regional CPE prevalence in England using the available surveillance data and, using these, calculated the expected introductions into each region to identify the largest CPE threats and assess the ability of admission screening on the basis of high-prevalence hospitals to prevent CPE introductions.

## Methods

### Hospital network structure

We calculated the ‘distance’ between hospitals in terms of patient movements (i.e. the number of times an inpatient is discharged from one hospital and then admitted to another) in the English National Health Service (NHS), to determine the most likely hospital-to-hospital transmission routes for pathogens such as CPE. We counted the number of exchanged in-patients between all pairs of hospitals (F_ij_) in the English NHS Hospital Episode Statistics (HES) for the financial year 2013–2014.

These patient exchanges could be direct transfers or indirect movements, where patients were discharged from one hospital to the general community and admitted to another hospital at a later time point that year. Using 1 year of HES data minimises variation in exchanged patients due to changes in the healthcare system structure. Throughout the analysis, we only included acute care hospital trusts and excluded any primary care, mental health or specialist trusts. Referral regions were defined based on transfers between these acute care hospital trusts, using a community assignment algorithm that maximises the modularity of the network [13]. Because these referral regions were defined based purely on the structure of the inter-hospital patient movement pathways, they do not necessarily overlap with administrative regions.

### CPE rates

As an approximate estimate of CPE prevalence, we used data collected through the national reference service of PHE’s Antimicrobial Resistance and Healthcare Associated Infections Reference Unit between 2008 and 2014. Clinical laboratories refer potential CPE isolates on a voluntary basis for confirmation and characterisation; there is no benchmarking or penalty from submitting isolates. There is no mandatory surveillance scheme for CPE in the UK, so these data were the best available on what is currently one of the largest AMR threats facing high-income countries. For each isolate, the reference laboratory determined the bacterial species and any carbapenem resistance mechanism found, and recorded the date of isolate receipt, the sampled source of the isolate (e.g. blood, sputum, rectal swab) and the sending laboratory. We coupled each sending laboratory to a hospital based on laboratory name and/or geographical location, or to a group of hospitals if it served a regional function. We categorised isolates by the carbapenemase family (KPC, OXA-48-like, NDM, VIM and IMP enzymes), irrespective of bacterial species, as CPE outbreaks may involve transposable-element-mediated spread of resistance genes across multiple species [14].

We calculated the CPE prevalence among discharged patients as the number of confirmed CPE isolates (N_r_) per 100,000 patient admission episodes (A_r_), thus using the incidence of confirmed isolates as a direct approximation of within patient population prevalence. We therefore assumed that any patient that was found to be CPE positive during its hospital stay was still colonised at discharge (and during a subsequent admission), as decolonising CPE positive patients is not advised (and is very difficult) [12].

We approximated the CPE prevalence (I_r,j_), both per resistance mechanism and for all CPE isolates for each region (j) defined as above. Admissions were determined using the in-patient admission records from the NHS-HES for the financial year 2013–2014, with A_r,j_ = ∑_h ∈ Hr_ A_h_, where H_r_ is the set of hospitals in region j.

See Additional file 1 for further details.

### Expected introductions

The number of admitted patients potentially colonised with CPE depends on the previous admission history of each newly admitted patient and the CPE prevalence in the hospitals they have visited. As CPE prevalence in most hospitals was low, to increase reliability in this exemplar, we considered the regional prevalence as the best approximation of the prevalence in all hospitals in the referral region. For each referral region, the number of expected introductions from all referral regions was calculated as P_i_ = ∑_j_ F_ji_ x I_r,j_, where F_ji_ is the connectedness between region *j* and *i*. We estimated the proportion of introductions from hospitals outside the region, from other hospitals within the region, and from the same hospital to determine the risk each posed to an individual hospital, and the effectiveness of import screening of patients from them, and related this to current guidance to focus screening only on ‘high-risk’ hospitals.

### Screening

We calculated the number of screens that would need to be performed if patients were selected based on previous admission to a ‘high-risk’ hospital using NHS-HES admissions during the financial year 2013–2014. We used three scenarios to trigger screening upon admission, (1) previous admission to the most-affected trust in the Manchester referral region, as a candidate of a single hospital that can be expected to be assigned as ‘high risk, (2) previous admission to any hospital in the Manchester referral region, to reflect a more diligent approach assigning an entire region, and (3) previous admission to any hospital in the London referral regions, to test the effect of including a region that consists of many hospitals.

## Results

**Fig. 1:**
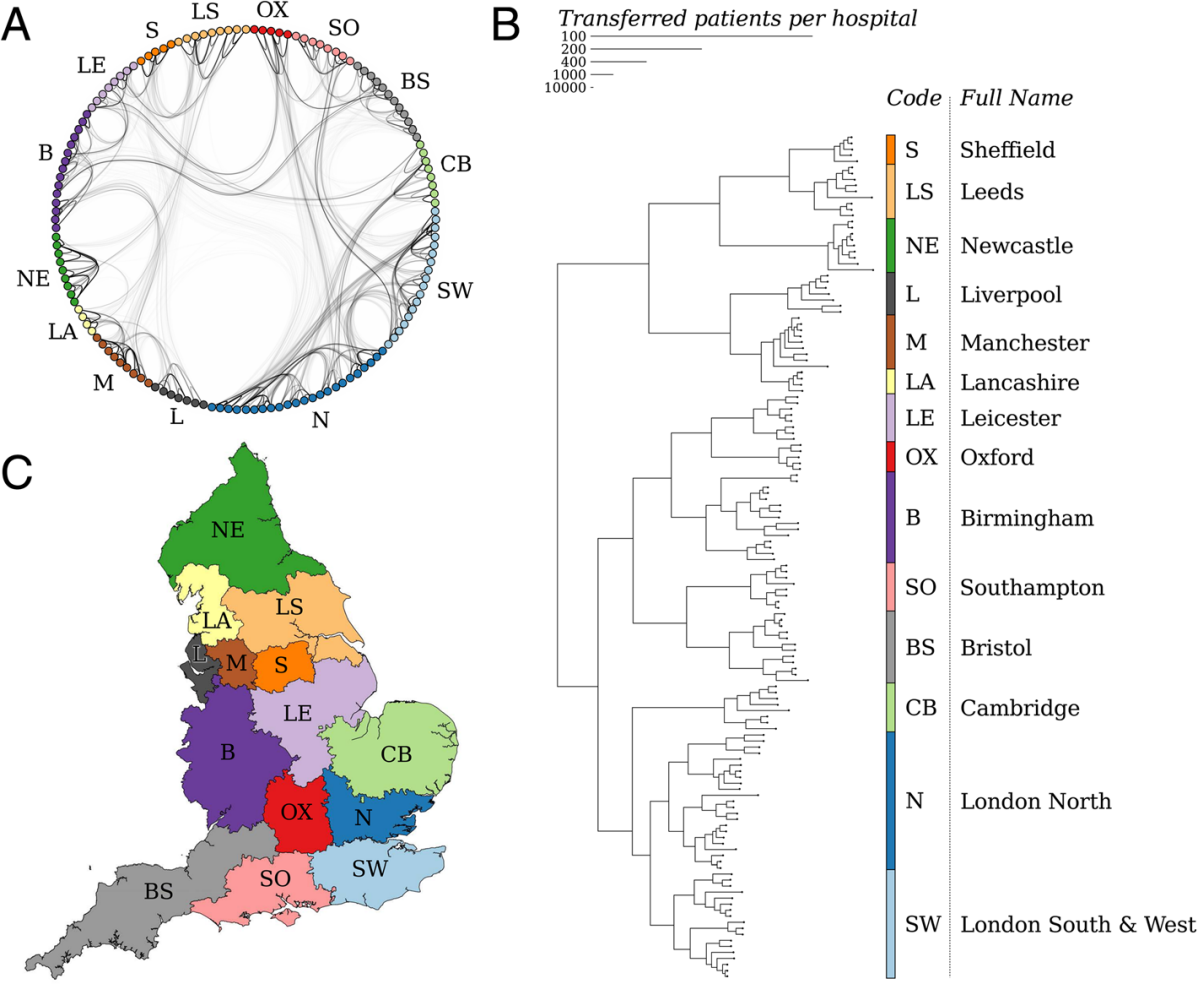
The structure of the patient referral network. **a** The network of all hospitals (*dots*) connected by exchanged patients (lines, darker lines indicating more exchanged patients) shows a clear regional structure. **b** The hospital network depicted by a neighbour-joining tree. The hospitals within each referral region exchange, with many more patients than hospitals in different regions. **c** The geographical distribution of the referral regions, based on the catchment populations of the hospitals

All hospitals in England were connected in one large network formed by patient movements (Fig. 1a), with a clear clustered structure. This network was divided into 14 groups of hospitals, denoted referral regions, using a standard community detection algorithm [13]. Hospitals within each region exchanged many more patients with each other than with hospitals in other regions (Fig. 1b). Despite the fact that these referral regions were purely based on the number of exchanged patients, hospitals within each referral region were geographically clustered (Fig. 1c). We therefore named the referral regions according to the largest city in each.

**Fig. 2:**
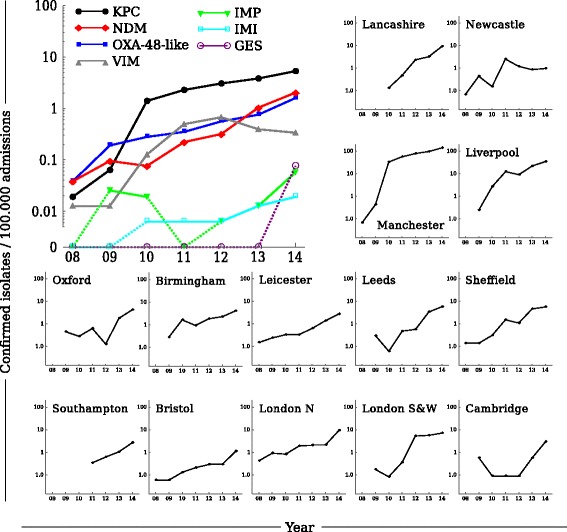
The incidence of confirmed carbapenemase-producing Enterobacteriaceae (CPE) isolates per 100,000 admissions, for England by resistance mechanism (large panel), and the total incidence of confirmed CPE isolates per referral regions in England (small panels), 2008–2014

The number of CPE isolates confirmed by the national reference laboratory increased sharply in all regions over 2008–2014 (Fig. 2) for all carbapenemase families, with KPC, NDM and OXA-48-like enzymes comprising most submitted isolates. Most of the clusters of confirmed isolates, signifying possible outbreaks, were confined to single hospitals (Additional file 1: Figure S1). However, a small number of apparent multi-institutional outbreaks were observed, the most obvious example of which was an outbreak of KPC-producers affecting hospitals in the Manchester referral region. Although the exact number of isolates from this referral region may have been higher due to the apparent stronger screening efforts (Additional file 1: Table S1), this regional outbreak contributed the vast majority of KPC-positive isolates confirmed by the reference laboratory (Additional file 1: Figure S1).

**Fig. 3:**
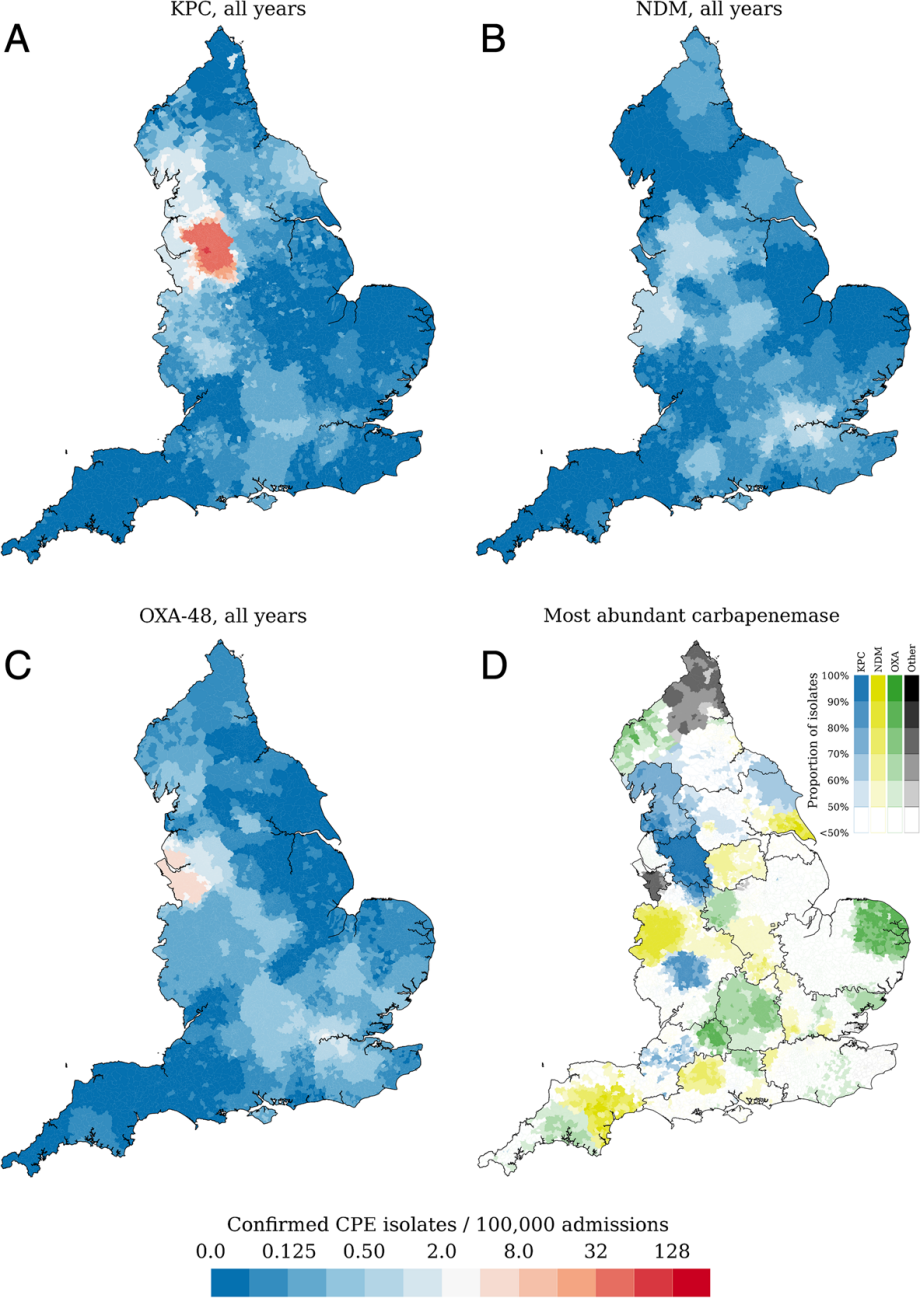
The geographical distribution of carbapenemase-producing Enterobacteriaceae isolates per resistance mechanism, calculated as the number of confirmed isolates per 100,000 hospital admissions (**a**–**c**) based on Hospital Episode Statistics data for 2013–2014. Clear differences can be discerned between mechanisms, with high prevalence of KPC and high OXA-48-like prevalence in different parts of the North-West. **d** The geographical distribution of the majority carbapenemase (colours) reflects both single hospital outbreaks as well as multi-institutional outbreaks, such as in the Manchester and Lancashire referral regions

These outbreaks were clearly visible in the geographical differences in both the CPE prevalence (Fig. 3a–c, Additional file 1: Figures S2–5) and the prevalent carbapenemases (Fig. 3d), with both NDM and OXA-48-like concentrated around London and the South and most KPC-positive CPE present in the North-West and the North. Despite its size, the Manchester outbreak has, to date, been largely confined to its own referral region, with far fewer confirmed isolates submitted from the Lancashire (n = 32) and Liverpool (n = 34) referral regions despite their proximity in the referral network (Fig. 1b).

If the most affected hospital in the Manchester region was the only trust on the high-risk list nationally, we estimated that 52,438 patients would need to be screened each year if the CPE toolkit recommendations (Table 1) were fully implemented; of these, 42,431 (80.9%) were newly admitted to other hospitals within the Manchester referral region. The same pattern was observed focussing on patients from hospitals in other referral regions with elevated CPE prevalence, such as London, where 88.8% (666,909) of the 750,939 patients eligible for screening (according to toolkit recommendations) were newly admitted to other hospitals within the London region.

**Table 1:** The number of patients to be screened per year, defined by the number of patients previously admitted to a ‘hospital with a known carbapenemase-producing Enterobacteriaceae (CPE) problem’ extracted from the Hospital Episode Statistics, under a number of assumed definitions of ‘problem hospitals’, readmissions to the same hospital were excluded

Hospitals with ‘known CPE problem’ defined as:	Screening patients admitted to:	Patients to be screened/year
Most affected Manchester hospital	*All other hospitals*	52,958
	*In same region*	42,431
	*Outside region*	10,527
All hospitals in the Manchester referral region	*All other hospitals*	228,575
	*In same region*	192,613
	*Outside region*	35,962
All hospitals in both London referral regions	*All other hospitals*	750,939
	*In same region*	666,909
	*Outside region*	84,030

**Fig. 4:**
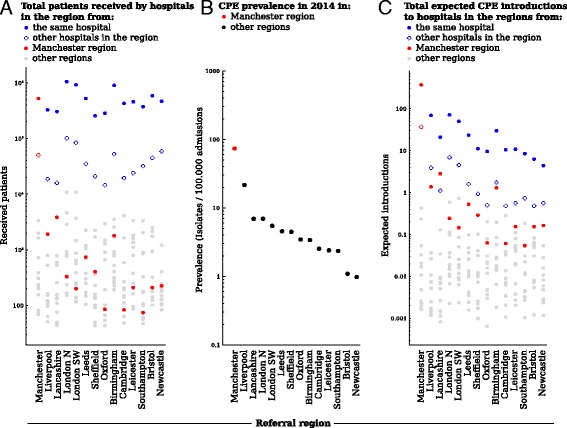
The number of patients received from each of the regions (**a**) multiplied by the prevalence in these regions (**b**) gives the total number of expected introductions into the current region (**c**). The difference in absolute number of expected introductions between regions is driven by patient flow, even if the relative prevalence differs considerably across the regions. **a** The number of patients received per region, from the same hospital (*solid blue dots*), other hospitals in the same region (*open blue dots*), hospitals in each of the other regions (*grey dots*), and hospitals in the Manchester region (*red dots*). **b** The prevalence of confirmed carbapenemase-producing Enterobacteriaceae (CPE) isolates in each region. **c** The expected CPE introductions per region. The example of the Manchester regions (*in red*) shows that the difference in absolute number of expected introductions between regions is primarily driven by patient flow, even if the relative prevalence differs considerably across the regions

**Fig. 5:**
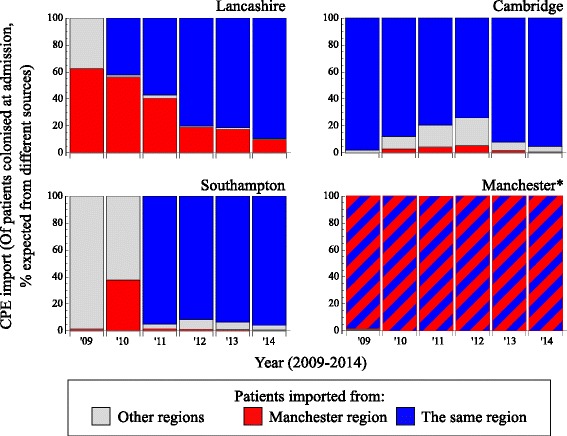
The number of expected introductions over time, based on the prevalence in each referral region in 2014, and the number of transferred patients from each region, showing all patients colonised at admission, with the expected proportion admitted from other regions (*grey*) and highlighting the contribution of patients coming from the Manchester referral region (*red*). The remaining proportion (*blue*) of received colonised patients (up to 100%) were previously admitted to hospitals in the same referral region, including the same hospital. Red/blue dashed bars are shown for the Manchester region (*), because patients from the same region and those from the Manchester region are the same

The difference in the number of patients who would need to be screened from within and outside the referral region is a direct reflection of the referral patterns. Because this difference in patient flow (Fig. 4a) is larger than the difference in prevalence (Fig. 4b), most hospitals can expect the majority of CPE introductions they experience to come through patients moving directly or indirectly from hospitals within their own referral regions (Fig. 4c). This effect was observed for hospitals in all regions, despite the large differences in prevalence between them. Hospitals in the Manchester referral region, for instance (Fig. 4, red dots), submitted 17 times more isolates per admission than hospitals in Lancashire, their closest neighbour in the patient referral network, yet only contributed 16.2% of the expected CPE introductions to Lancashire hospitals over 2008–2014 (Fig. 5a). Overall, 89.5% of expected introductions originated from the same hospital, 8.7% from the same referral regions, and the remaining 1.8% from other referral regions.

Generally, the number of patients received from hospitals inside the referral region, including those patients previously admitted to the index hospital, was a hundred-fold greater than the number received from hospitals outside the region. If only patients coming from other hospitals within the region are considered, thus excluding readmissions from the same hospitals, there was still a ten-fold difference between the patient flow within and between regions (Additional file 1: Figure S6). The prevalence in other regions therefore needed to be two orders of magnitude higher than within the region for cross-regional introductions to be equal to the introductions from within the region (Additional file 1: Figure S6).

Finally, we considered how the interplay between prevalence and patient movements varied over time as the new AMR threat became entrenched. As the CPE prevalence in all regions increased over time (Fig. 2), the relative importance of introductions from hospitals outside the referral region in fact decreased (Fig. 5). Considerable numbers of expected introductions from other referral regions were only seen initially in regions with a low CPE prevalence at that time (e.g. Southampton) or that bordered a region with high CPE prevalence (e.g. Lancashire). The strong influence of the increasing within-regional prevalence therefore makes cross-region screening less effective as time goes by.

## Discussion

To combat the spread of AMR, hospitals are advised to screen high-risk patients for carriage of antibiotic-resistant bacteria on admission to limit opportunities for onward transmission. One commonly used risk factor is previous admission to a hospital with a known resistance problem, such as a current outbreak, or high endemic prevalence of resistant organisms. Screening patients from such ‘high-risk’ hospitals will identify the largest proportion of colonised patients per screened patient because of the greater relative prevalence in ‘high-risk’ hospitals. However, in terms of the risk of introduction and subsequent transmission to new patients, it is the absolute number of colonised patients received by a hospital which determines the chance of other patients becoming colonised. For example, if 50% of two patients received from a high-risk hospital are colonised, this one patient poses a lower onward transmission risk if unidentified than 1% colonised patients out of 1000 patients previously admitted to the same hospital (10 patients). The structure of the patient referral network dictates that not all hospitals pose an equal risk to others because patient exchanges are not randomly distributed. As larger outbreaks are more likely to draw more attention, even if they occur further away, this can result in a discrepancy between the perceived and actual risk of receiving colonised patients.

To illustrate this, we considered, as an exemplar, CPE introductions into NHS hospital trusts in different referral regions in England. Substantial increases in CPE over the period 2008–2014 reflect what would be expected for a new AMR threat, particularly one initially predominantly confined to healthcare settings. Overall, CPE has made alarming gains in England over the last decade, with many cases forming part of larger clusters as opposed to individual introductions. Although many of the single CPE isolates found in various hospitals may still be the result of introductions from abroad [15, 16], the spread of CPE within English hospitals is gaining importance in the overall epidemiology. Our results should therefore be broadly generalisable to future AMR threats.

In contrast with the common practice of import screening based on assigning a few hospitals as high risk, we found that transfer of patients between hospitals with small AMR problems in the same region often pose a larger threat in terms of absolute numbers of colonised patients admitted to a hospital than transfer of patients from hospitals in other regions, even if the prevalence of AMR in the other regions is orders of magnitude higher. This means that, with the current pattern of hospital referrals, the closeness of other hospitals is a much greater driver for the spread of antibiotic-resistant bacteria than their AMR prevalence. As a rule of thumb, for any given referral region, the prevalence of AMR in another referral region needs to be at least 100-times higher to contribute substantially to the admission of patients colonised with antibiotic-resistant bacteria; equivalently, without knowledge of the regional structure, hospitals which share most patients pose a greater threat than those with the greatest prevalence, unless there are marked disparities in prevalence.

An outbreak of KPC-positive Enterobacteriaceae limited to hospitals in the Manchester referral region demonstrated the multi-institutional component of CPE dispersal, largely driven through patient movements. The observation that hospitals in the Liverpool referral region were less affected by KPC-positive bacteria, despite their geographical proximity to Manchester, illustrated the lower risk of cross-regional introduction. Screening of relatively few patients could therefore potentially be used to mitigate inter-regional spread at the start of an outbreak, although our results suggest this would become less effective with time. Such compartmentalisation only prolongs the time to a successful introduction [17] because the chance remains that the admission of one undetected colonised patient may seed a new outbreak. Any inter-regional screening efforts should therefore always be accompanied by intra-regional or hospital-specific control efforts as our results show that this is often the most likely initial source of resistant micro-organisms.

Several studies have highlighted the necessity of coordinating IPC activities within regions [18], often building on the structure of the patient referral network [19–21]. This can, for example, be done by sharing information about the colonisation status of patients between healthcare institutions, through centralised registers [22] or by patient held cards [23], alerting hospitals to take appropriate action to prevent onward transmission and saving money from unnecessary repeated screening. Regional coordination may also aid effective contact tracing in outbreaks that span multiple institutions. Our study shows that it is essential for hospitals to get an up-to-date overview of the true prevalence of CPE, or AMR in general, in their surrounding hospitals to estimate the risks they pose through shared patients. The improved sharing of prevalence estimates, for instance obtained through periodic point-prevalence surveys for AMR, between all healthcare trusts, and in particular those referring many patients, should therefore be promoted.

We did not find evidence that the increase in CPE over the last decade was due to large-scale breakdowns in IPC standards, again suggesting our results should be broadly applicable to new AMR threats arising on a background of ongoing IPC efforts. The single isolates or small outbreaks of CPE affecting hospitals throughout the country represented many carbapenemase types, but hospitals with longer outbreaks usually only ‘suffered’ from a single dominant carbapenemase and did not have parallel outbreaks involving multiple carbapenem resistance mechanisms despite what appears to be continuous new exposures. However, it is possible that temporary lapses in IPC give any AMR threat the opportunity to spread unseen after introductions, giving rise to larger outbreaks and highlighting the importance of continued vigilance. Moreover, the length of some of these outbreaks indicate that they may be hard to eradicate once established. This could be caused by colonised patients returning to hospital, thus reintroducing the bacteria repeatedly, or the establishment of environmental AMR reservoirs within the hospital.

The main study limitation is that we were only able to approximate the prevalence of our exemplar, CPE, since data came from a voluntary system of submitting isolates to a reference laboratory and reporting rates may differ considerably between hospitals and over time as screening efforts change depending on the perceived problem. However, firstly, the overall increase in CPE nationally is unlikely to be solely the result of changes in screening practice, and regional differences in the occurrence of the different resistance mechanisms should not be affected. Secondly, it is likely that hospitals with a known CPE problem, such as those in the Manchester referral region, engaged in a more active search for possible colonised patients, apparent from the higher proportion of isolates originating from rectal swabs, and therefore submitted relatively more isolates. If anything, this would have caused the differences between regions and the effect of reported problems further away to be over-estimated, which means, in turn, that the contributions from hospitals within the referral region are probably even greater than we estimate. Finally, the goal of this study was to investigate the interplay between hospital networks and recommendations for dealing with new AMR threats that have generally focussed on screening patients from ‘high-risk’ hospitals because of their greater relative prevalence. We investigated CPE only as an exemplar of such a new infection threat – our conclusions do not depend on the specific organism, only on variation in prevalence across regions and changes in its distribution being broadly what one would anticipate from a new AMR threat, and the main current method of transmission being hospital based.

Our calculations were based on the structure of the inter-hospital patient referral pathways. This implicitly assumes that, for the organism in question, community spread of AMR occurs rarely, and leaves out other healthcare facilities, such as long-term care facilities or nursing/residential homes. If community acquisition becomes an important part of the dispersal mechanisms, the regional referral networks may become less meaningful, since community acquisition would impose an additional relatively uniform probability of introduction on each hospital, diluting the effect of import screening from other hospitals, irrespective of how far away they are in the network. However, it is likely that contact patterns between patients in other healthcare institutions, or other social structures or community interactions, reflect the structure of the patient referral pathways. Furthermore, the observed regional differences in CPE resistance mechanisms do suggest that, at present, the dispersal of this AMR threat is primarily taking place within the healthcare regions.

We were unable to estimate the introductions by patients previously admitted to foreign hospitals due to the lack of reliable admission numbers. However, it is unlikely that the number of admissions from abroad would surpass the number of admissions from other regions in England. We would therefore tentatively suggest that the same conclusion applies to patients from abroad, namely that the number of introductions from abroad only contributes considerably if the prevalence within the region is orders of magnitude lower than the prevalence in the other country.

While we treated all exchanged patients as being equally likely to be colonised, the risk of colonisation will differ depending the ward/unit they were admitted to and/or their underlying health status. Although this may alter the exact risk posed by exchanged patients, the risk posed by far away hospitals relative to neighbouring hospitals is unlikely to increase dramatically if patient-specific risks are taken into account given the large difference in patient flows within and between regions. Adjusting the screening policy to differentiate patients based on the ward of current admission might be feasible [24], however, it would be more difficult to rely on information about the visited ward during the previous hospital visit.

## Conclusions

The dispersal of AMR, particularly CPE, calls for immediate concerted action. To combat AMR effectively, IPC measures need to be coordinated, as it is no longer a problem for single hospitals but of the entire healthcare system. We have shown that it is important to control the dispersal of AMR on a local or regional level, rather than focussing on large problems far away, because the expected absolute number of colonised patients received from hospitals within the region is often far greater than those received from other regions, even if the prevalence in those regions is orders of magnitude higher. The primary concern in the assessment of the risk posed by each hospital, needed to inform IPC measures, is the availability of reliable data on AMR prevalence in each hospital. Hospitals should therefore be encouraged to undertake periodic point-prevalence surveys for AMR in general, and CPE in particular, to estimate their true prevalence, and to share this information with the other hospitals in their referral region.

## Additional file

Additional file 1: Additional methods and species-specific inceidence maps. (DOC 3368 kb)

## Abbreviations

AMR: antimicrobial resistance
CPE: carbapenemase-producing enterobacteriaceae
HES: hospital episode statistics
IPC: infection prevention and control
KPC: Klebsiella pneumoniae carbapenemase
MRSA: methicillin-resistant Staphylococcus aureus
NDM: New Delhi metallo-beta-lactamase
NHS: National Health Service
OXA-48: carbapenem-hydrolysing oxacillinase-48
VIM: Verona integron-encoded metallo-beta-lactamase
