# Supplementary material for: The relative importance of large problems far away versus small problems closer to home: insights into limiting the spread of antimicrobial resistance in England
Source: BMC Med. 2017 Apr 27;15:86. doi: 10.1186/s12916-017-0844-2 (PMC5406888; doi:10.1186/s12916-017-0844-2)
Supplement: Additional file 1: — Additional methods and species-specific inceidence maps. (DOC 3368 kb) [file 12916_2017_844_MOESM1_ESM.doc]

**The relative importance of large problems far away vs. small problems closer to home: insights into limiting the spread of antimicrobial resistance in England**

***Supplementary text***

*Mapping*

We determined the CPE incidence (I), both per resistance mechanism and for all CPE isolates, as the number of submitted CPE isolates (N) per 100000 admissions (A), for each hospital (h) and per region, including all laboratories assigned to specific hospitals within a region (r) and including the laboratories in a region but not assigned to a specific hospital (l). The number of admissions per hospital trust was determined using the in-patient admission records from the National Health Service in England (NHSE) hospital episode statistics (HES) for the financial year 2013-14:

I_h_ = N_h_ / A_h_,

I_l_ = N_l_ / ∑_h_ _∈ Hr_ A_h_,

I_r_ = N_r_ / ∑_h ∈ Hr_ A_h_,

where H_r_ is the set of hospitals in region r, N_r_ is the number of isolates submitted to laboratories assigned to specific hospitals within a region r, and N_l_ is the number of isolates assigned to laboratories in referral region that were not assigned to a specific hospital.

Hospital catchment areas were determined based on the electoral ward of residence of the patients, using the NHSE HES. For each electoral ward (w) we determined the proportion of patients going to each hospital (p_wh_) and each referral region of hospitals (p_wr_) to calculate the weighted average incidence (W_w_) over all hospitals. The electoral wards' centroids were used to create a smoothed contourplot of the geographical distribution of CPE incidence,

W_w_ = ∑_h_ (p_wh_ I_h_) + ∑_r_ (p_wr_ I_l_).

As well as mapping the geographical distribution of carbapenemase types, we also mapped the distribution of carbapenemase types per bacterial genus (Figure S2-S5). Isolates from different bacterial genera with the same carbapenemases clustered geographically, indicating that the carbapenemases are plausibly spreading between species, most likely through exchange of transposable elements, reaffirming the importance of studying the carbapenemases as the unit of spread in this exemplar application, instead of the individual bacterial species containing the specific carbapenemases.

*Risk tree structure*

Throughout the analysis, we only included acute care hospital trusts and excluded any primary care, mental health or specialist trusts. Referral regions were defined based on transfers between these acute care hospital trusts, using a community assignment algorithm that maximises the modularity of the network^1^. Because these referral regions were defined based purely on the structure of the inter-hospital patient movement pathways, they do not necessarily overlap with administrative regions.

This implicitly assumes that community spread of CPE does not occur (or at least occurs very infrequently relatively to hospital spread), and leaves out other healthcare facilities, such as long-term care facilities and nursing/residential homes. If community acquisition becomes an important part of the dispersal mechanisms, the regions based on the referral networks may become less meaningful, although these are still likely to reflect other social structures or community interactions. However, the large difference in CPE incidence between North West and its neighbouring regions, together with the observed regional differences in resistance mechanisms, indicate that at present the dispersal is primarily taking place within the healthcare regions.

A high degree of regional structuring of the patient population was clearly shown when using patient transfers to estimate connectivity between different NHS hospitals (Figure 1). Most readmitted patients were previously admitted to the same hospital (90.5%); patients transferred between hospitals predominantly moved within their referral region (7.6%), and to a much lesser extent to hospitals in other referral regions (1.9%). The rate of readmissions was very high, with 52.5% of admissions occurring in patients who had been admitted to hospital in the last year. A large proportion of these re-admissions (54.4%) were due to a small group (5.0%) of frequently attending patients, with more than five admissions per year, often to the same hospital.

*Cluster assignment*

Isolates carrying a specific family of carbapenemase were grouped into clusters if the time between their submission to the reference laboratory was shorter than could be expected by chance, so that each cluster could be considered the result of an independent introduction. Based on a Poisson process, the expected time between receiving two isolates is exponentially distributed, with a mean of *1/m*, where m is the rate of introduction, the number of introductions divided by the total time (hospitals x years): *m = n / (h * Y)*. Isolates were considered to belong to the same cluster if the time between receiving them was shorter than the 5^th^ percentile expected according to the exponential distribution (*T<Log(0.95) / m*). The initial m was determined by treating all isolates as introductions, and isolates were then clustered based on the threshold *T*. The process was then iteratively repeated treating each cluster, as well as the remaining independent isolates, as single introductions to calculate a new rate *m* and threshold *T*, until the number of introductions remained the same.

No information was available on the actual dates isolates were taken, and we therefore used the dates when the national reference laboratory received the isolates. As some laboratories may have chosen to send isolates in batches, a number of isolates could have been falsely assigned to a cluster based on the timing of their submission. However, the resulting potential underestimation of the number of independent isolates does not influence our final conclusions. Given the importance of identifying CPE ‘outbreaks’ to submitting hospitals, it is not likely that there were long delays in submitting large numbers of isolates.

1 Clauset A. Finding local community structure in networks. Phys Rev E 2005; 72: 1–6.

Table S1: The source of isolates per referral region. For most isolates, the sample source of the isolate was provided. The high proportion of isolates from rectal swabs in Manchester and Leeds point towards stronger screening efforts, although it cannot be said with absolute certainty which isolates were the result of screening efforts or taken because of clinical necessity. *Numbers for both London referral regions were combined.


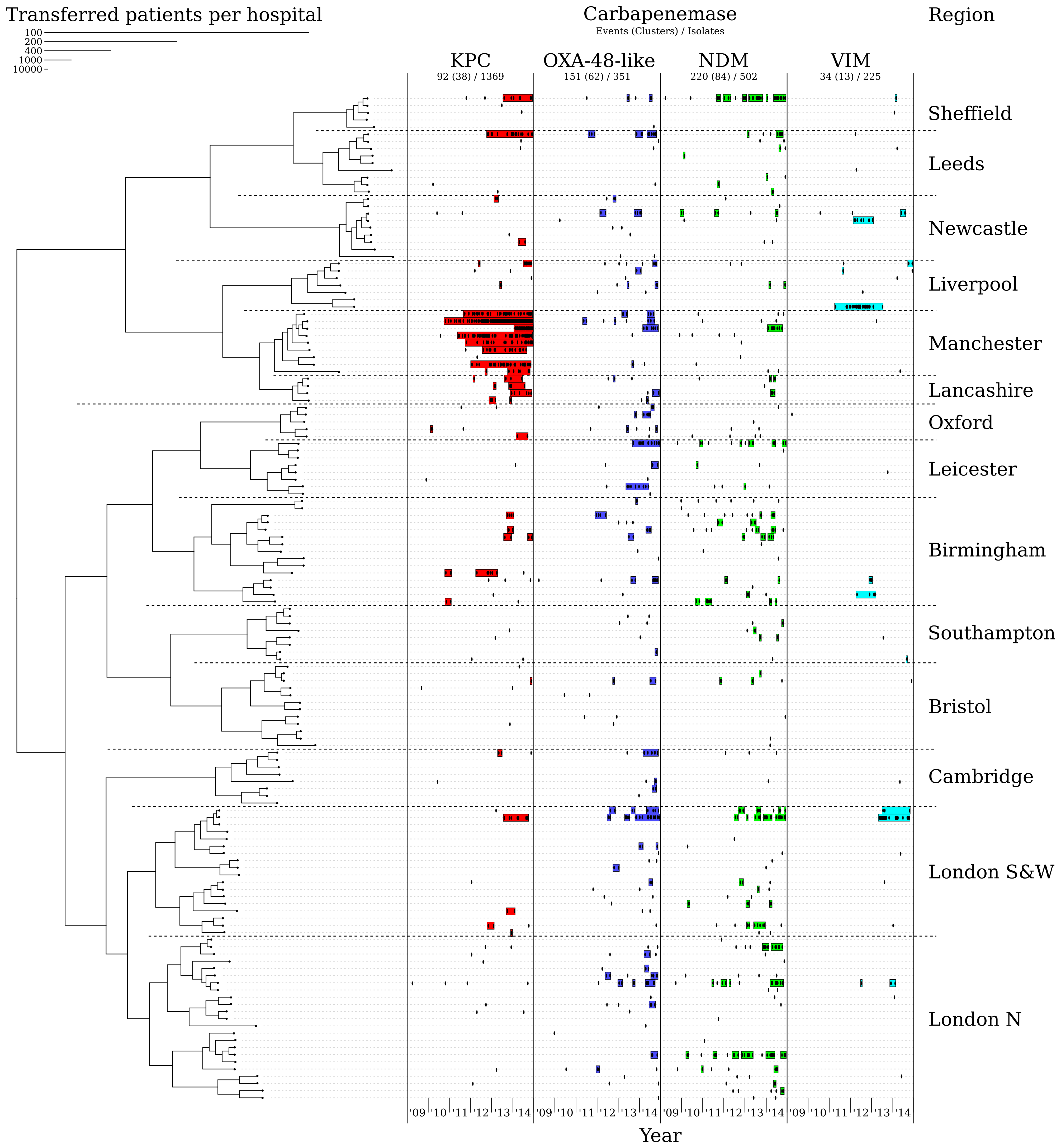


Figure S1: The structure of the patient referral network, depicted by a neighbour-joining tree based on the number of transferred patients in the 2013-2014 financial year. Each leaf on the tree represents a single hospital trust and branch lengths (scale on top left) denote the mean number of transferred patients between the two groups. The submitted isolates (right side, dots) were divided into single introductions (dots) and clusters (coloured bars), determined by comparing the time between receiving two isolates and the expected time based on the average rate of introduction for that carbapenemase (see supplementary methods).


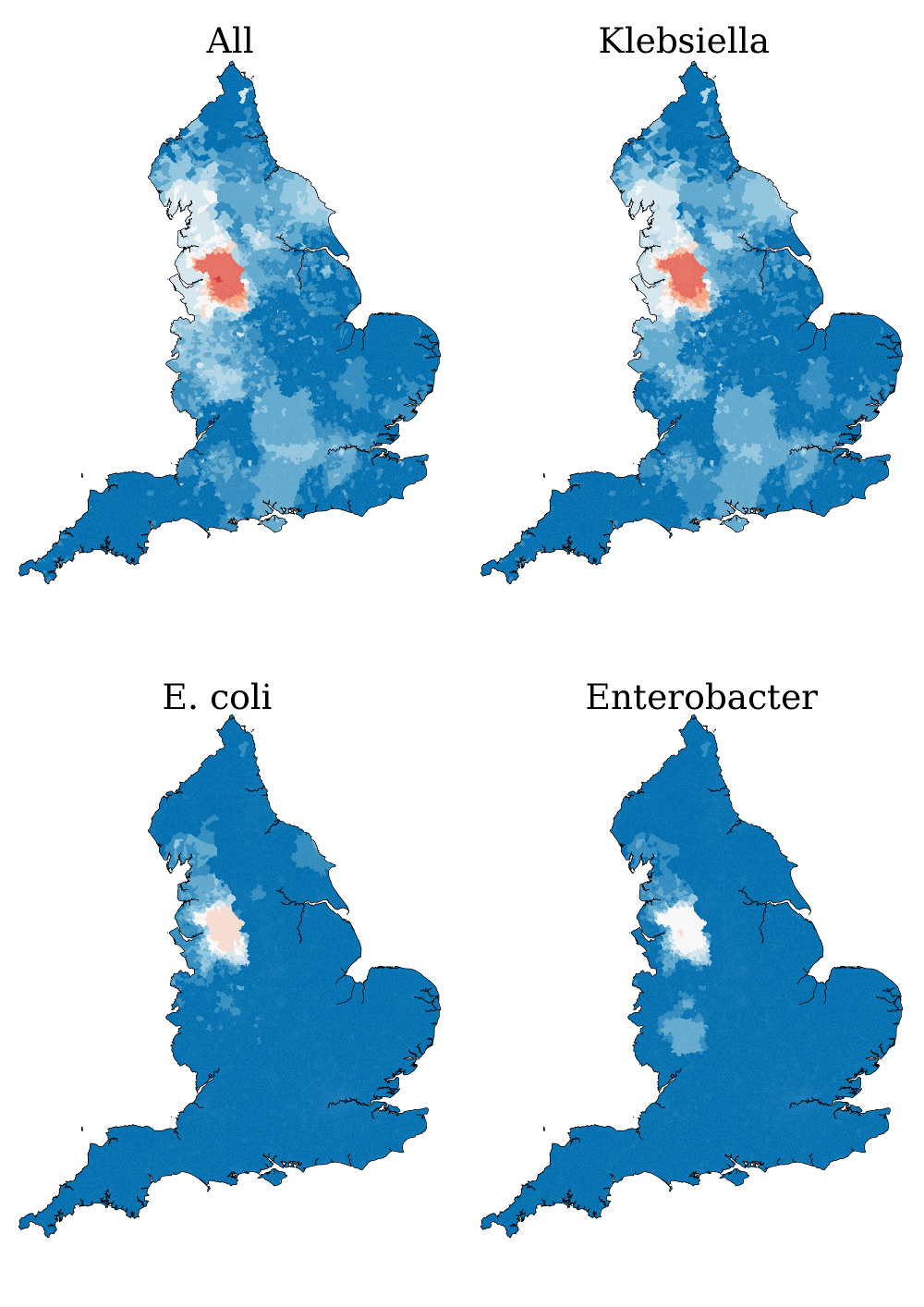
Figure S2: The geographical distribution of KPC positive isolates per bacterial genus, calculated as the number of submitted isolates per 100,000 hospital admissions.


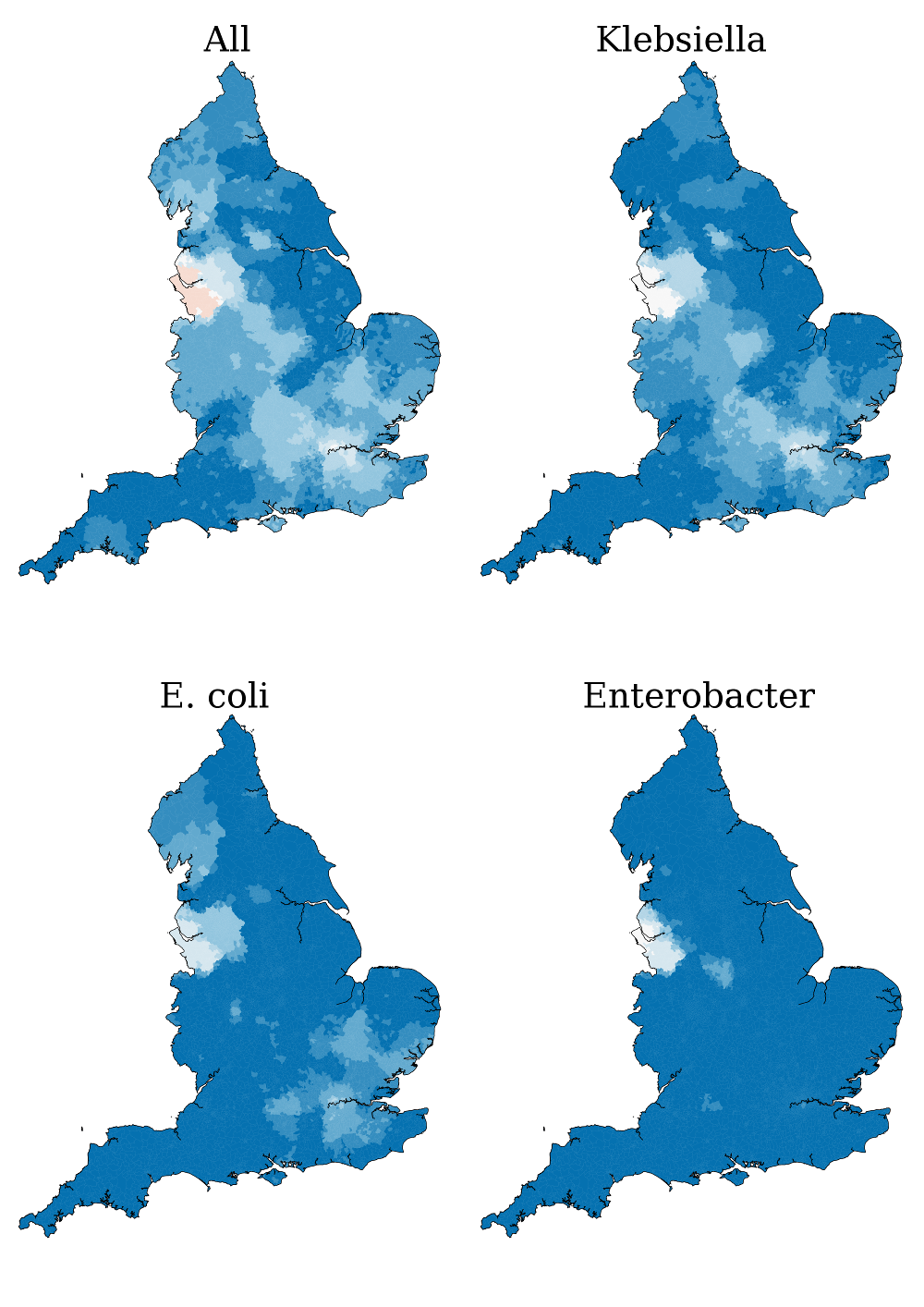


Figure S3: The geographical distribution of OXA-48-like positive isolates per bacterial genus, calculated as the number of submitted isolates per 100,000 hospital admissions.


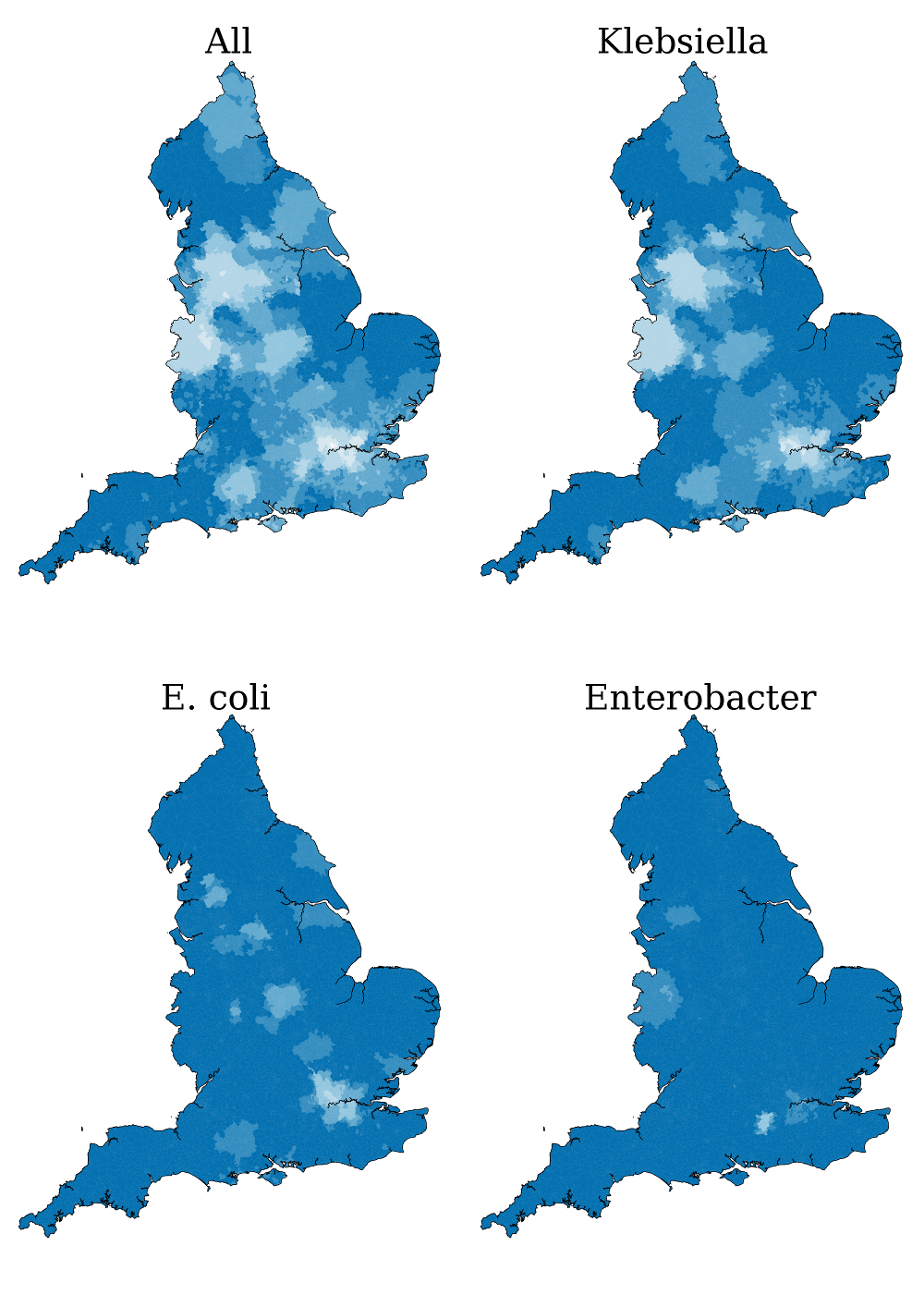


Figure S4: The geographical distribution of NDM positive isolates per bacterial genus, calculated as the number of submitted isolates per 100,000 hospital admissions.


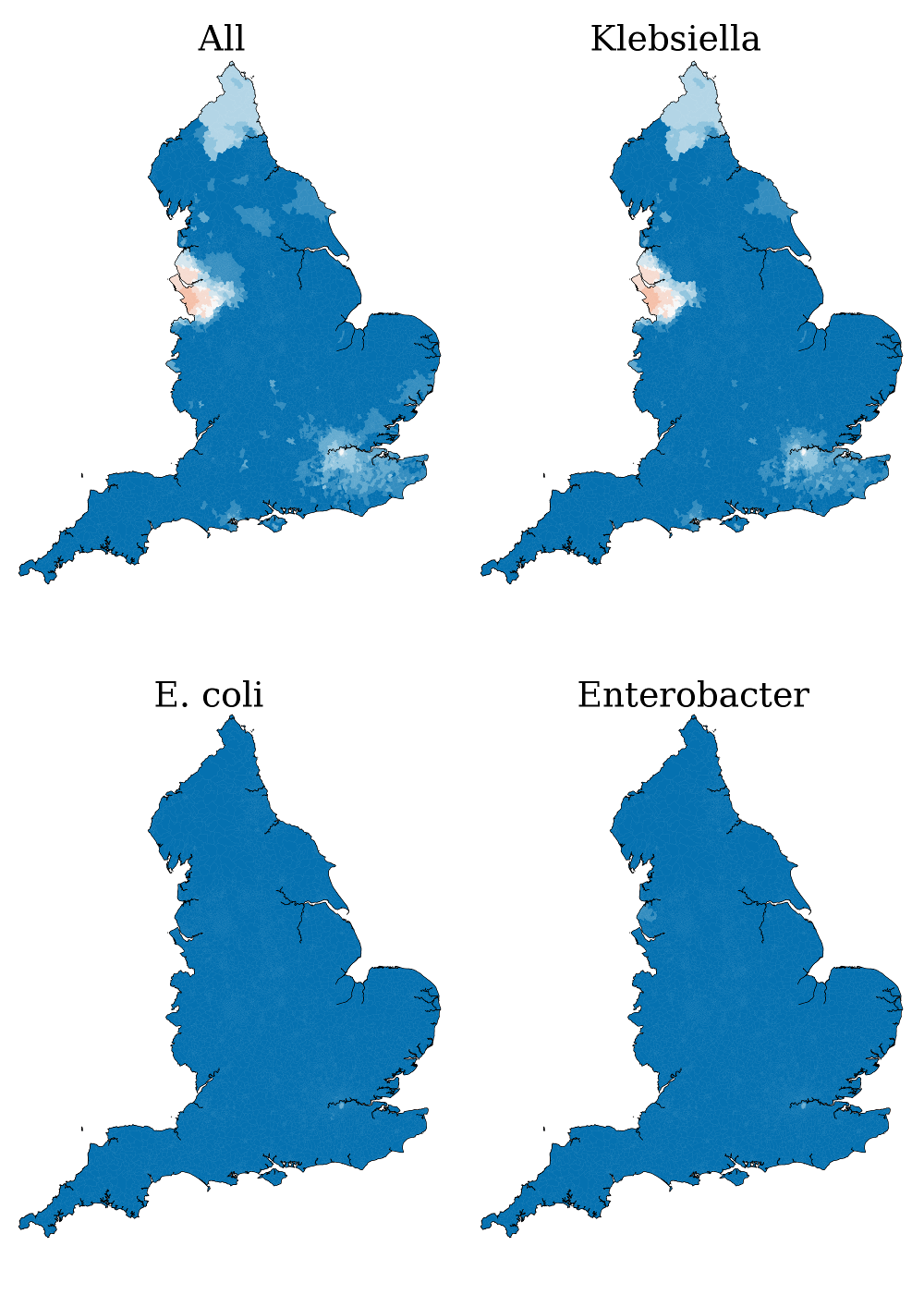


Figure S5: The geographical distribution of VIM positive isolates per bacterial genus, calculated as the number of submitted isolates per 100,000 hospital admissions.


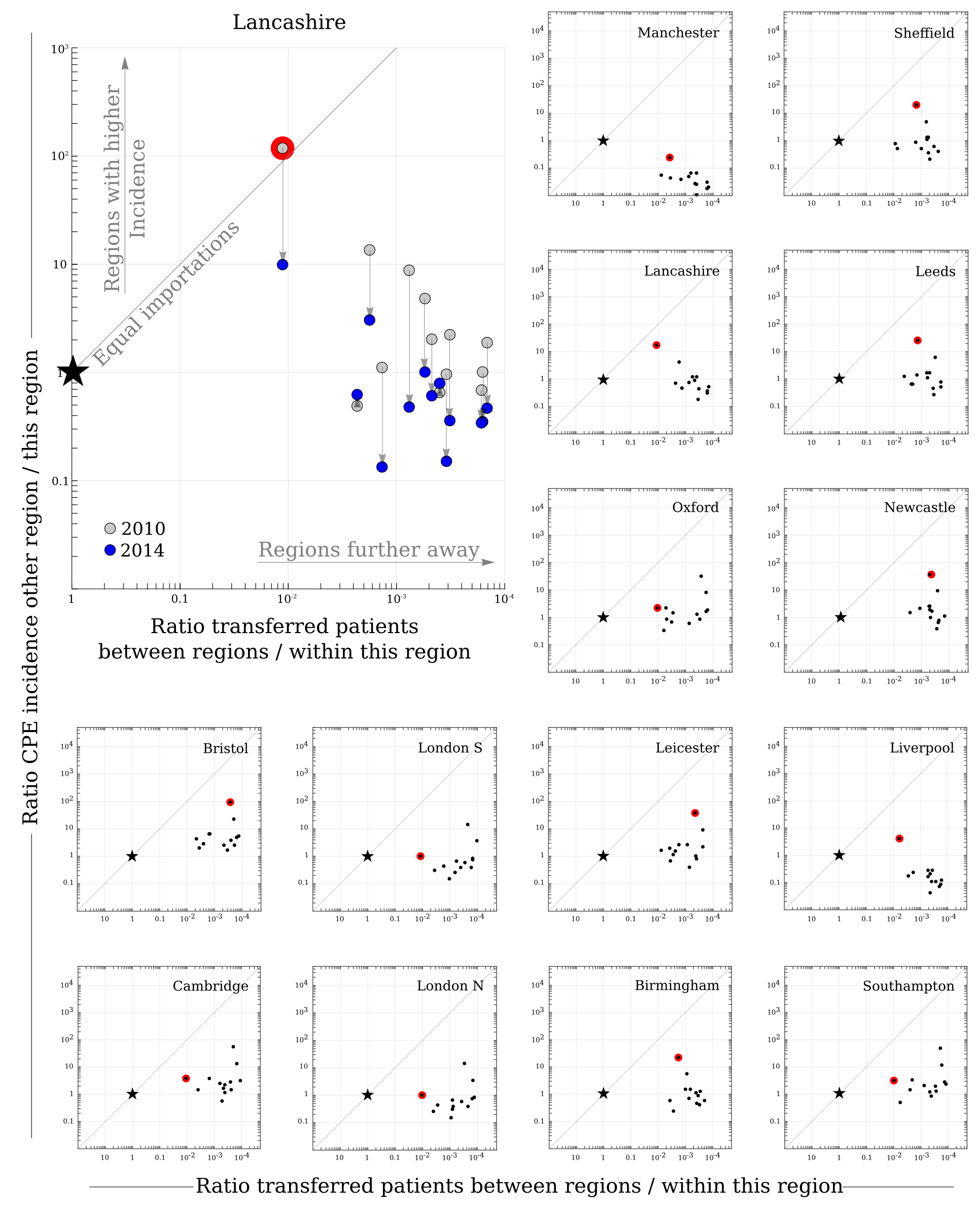


Figure S6: The panels show the relative importance of the incidence of CPE and the number of patient movements between referral regions for each referral region. The X-axis shows the distance in terms of number of patient movements between the regions, with regions exchanging fewer patients between the named index region further to the right on the x-axis (i.e. inverse to usual axis orientation, explained below). The Y-axis shows the difference in CPE incidence between the index region and other regions, with high-incidence regions higher. Each dot shows the difference in exchanged patients and CPE incidence between the index region (named in the plot subtitle) and one other region. The star represents the index region, and is therefore always located at (1,1) because the x-axis and y-axis show transfers and incidence relative to the index region. The diagonal line through this point represents a region where an equal **number** of CPE colonised patients are received from within the index region as from the other region. This can happen because a small number of patients are exchanged with a region with high incidence (top right) or a large number of patients are exchanged with a region with low incidence (lower left). Any points above the line are thus regions that contribute significantly to the CPE import in the index region. The red dots indicate the region that acts as the major contributor for imported cases into each region (closest to the diagonal line). The large panel shows the development over time for the Lancashire region, using the incidence for 2010 (gray) and 2014 (blue). One region was expected to contribute significantly to the introductions into Lancashire hospitals in 2010, but this effect diminished over time.
